# *Petrodesmum*, a new genus of the legume tribe Desmodieae from Laos

**DOI:** 10.1186/s12870-026-08552-4

**Published:** 2026-03-12

**Authors:** Kai-Wen Jiang, Jun Zhang, Shi-Jin Li, Zhu-Qiu Song

**Affiliations:** 1 Key Laboratory of National Forestry and Grassland Administration On Plant Conservation and Utilization in Southern China, South China Botanical Garden, Chinese Academy of Sciences, Guangzhou, 510650 China; 2 Ningbo Botanical Garden, Ningbo, 315201 China; 3 Herbarium, Bazi Collection and Botanical Garden, Mengzi, 661100 China; 4 Kunming Zhifen Biotechnology Co., Ltd., Kunming, 650504 China

**Keywords:** *Desmodium*, Fabaceae, Leguminosae, New genus, Phylogeny, Taxonomy

## Abstract

Recent molecular evidence has revealed that *Desmodium* (Fabaceae: Desmodieae), as traditionally defined in *Legumes of the World*, is highly polyphyletic and has now been subdivided into 18 distinct genera. The currently circumscribed *Desmodium* is primarily distributed in the Americas, with only one species native to Africa and none naturally occurring in Asia. Approximately one-third of the Asian species formerly classified under *Desmodium* have been reassigned to the recently resurrected genus *Grona*. The Laotian species *Desmodium vidalii*, which was originally placed in *Desmodium* subg. *Podocarpium* (now treated as *Hylodesmum* and *Monarthrocarpus*), was recently transferred to *Grona*. However, this species exhibits a suite of distinctive morphological and ecological traits—such as a herbaceous habit with short zigzag stems, leaves bearing an exceptionally long petiole and a long rachis of the terminal leaflet, long laxly flowered inflorescences, deeply constricted pods with a stipe, and occurrence in limestone habitats—suggesting that its generic placement requires re-evaluation. Using plastid and nuclear ribosomal DNA sequences, as well as 353 single-copy nuclear genes, and based on comprehensive sampling of all 47 genera and approximately 32% of species in the tribe, our phylogenetic analyses recognize 20 major clades within Desmodieae. *Desmodium vidalii* constitutes an independent clade, clearly separate from *Grona*, *Hylodesmum*, and *Monarthrocarpus*. Consequently, we propose the new genus *Petrodesmum* K.W.Jiang & Z.Q.Song to accommodate this species. A key to all 29 genera of Desmodieae occurring in Laos is also provided.

**Supplementary Information:**

The online version contains supplementary material available at 10.1186/s12870-026-08552-4.

## Introduction

Desmodieae (Benth.) Hutch. is recognized as one of the most highly specialized tribes within the subfamily Papilionoideae (Fabaceae/Leguminosae) [24]. The tribe comprises approximately 550 species, predominantly shrubs or subshrubs. These are distributed across tropical and subtropical regions worldwide, with the highest species richness in Asia (ca. 262 species), followed by the Americas (ca. 188 species), Australia (ca. 72 species), and Africa (ca. 66 species) [4]. Early classifications divided the tribe into 27 genera across three subtribes (Bryinae, Lespedezinae, and Desmodiinae) based on morphological evidence [24]. This system was later revised using molecular data from the plastid *rbcL* gene, which excluded the subtribe Bryinae and reclassified the remaining species into three informal groups within two subtribes: the *Lespedeza* group of Lespedezinae, and the *Phyllodium* and *Desmodium* groups of Desmodiinae [16].

As the core member of the *Desmodium* group, *Desmodium* Desv. was established by Desvaux [2] based on four American species and one Asian species, with the American *D. scorpiurus* (Sw.) Desv. designated as its conserved type (see [39]). In the *Legumes of the World*, Ohashi [16] recognized about 275 species in the genus, noting its greatest diversity in Southeast Asia (at infrageneric level) and in Mexico to South America (at species level). However, subsequent molecular phylogenetic studies demonstrated that this broadly defined *Desmodium* was highly polyphyletic, prompting a comprehensive revision. This led to the establishment of a new classification comprising 18 genera, including 13 new and 4 resurrected genera [25–27, 29, 31]. These segregated genera largely correspond to the subgenera, sections, subsections, or series previously recognized within *Desmodium* by Ohashi [14] and Pedley [34]. Among these, the resurrected genus *Grona* Lour. has subsequently expanded to become the second-largest genus within Desmodieae, comprising 49 species worldwide, distributed across Asia (24 species), Africa (17 species), Australia (12 species), and the Americas (3 species) [4, 22, 32]. Under this new framework, the Asian species formerly placed in *Desmodium* have been redistributed among 12 distinct genera, with approximately one-third transferred to *Grona* (see Table 1).

**Table 1:** New and resurrected Asian genera segregated from *Desmodium* sensu Ohashi [16]

Genus	Species number	Distribution	Reference
*Bouffordia* H.Ohashi & K.Ohashi	1	Asia, Australia	[25]
*Grona* Lour	49	Asia (24 spp.), Australia, Africa, South America, North America	[19]
*Huangtcia* H.Ohashi & K.Ohashi	2	Asia	[25]
*Murtonia* Craib	1	Asia	[30]
*Ototropis* Nees	8	Asia	[28]
*Oxytes* (Schindl.) H.Ohashi & K.Ohashi	4	Asia (1 sp.), Australia	[25]
*Pleurolobus* J.St.-Hil.	6	Asia (3 spp.), Australia, Africa	[25]
*Polhillides* H.Ohashi & K.Ohashi	1	Asia, Africa	[27]
*Puhuaea* H.Ohashi & K.Ohashi	2	Asia	[28]
*Sohmaea* H.Ohashi & K.Ohashi	7	Asia	[21]
*Sunhangia* H.Ohashi & K.Ohashi	6	Asia	[28]
*Tateishia* H.Ohashi & K.Ohashi	3	Asia	[25]

*Desmodium vidalii* H.Ohashi was originally described from a single specimen collected in central Laos [15]. In the protologue, it was placed within *Desmodium* subg. *Podocarpium* Benth. This subgenus was subsequently split into two distinct genera, *Hylodesmum* H.Ohashi & R.R.Mill and *Monarthrocarpus* Merr. [17, 40]. However, *D. vidalii* was not assigned to either genus at that time. Instead, it was recently transferred to *Grona* by Ohashi and Ohashi [23]. This revised classification is based on their comprehensive assessment of multiple morphological characters, which collectively indicated that the species possesses intermediate features between the aforementioned genera. Morphologically, as described [15, 23], this species exhibits a suite of distinctive traits, such as an herbaceous habit with short zigzag stems, leaves bearing an exceptionally long petiole and a long rachis of the terminal leaflet, laxly flowered long inflorescences, and deeply constricted pods with a long stipe. Furthermore, its occurrence in the fissures of limestone rock walls is unique within the tribe Desmodieae [15]. Given its intermediate morphology and unique ecology, the current generic placement of *D. vidalii* requires re-evaluation, particularly in light of molecular data.

## Materials and methods

### Morphological studies

**Fig. 1:**
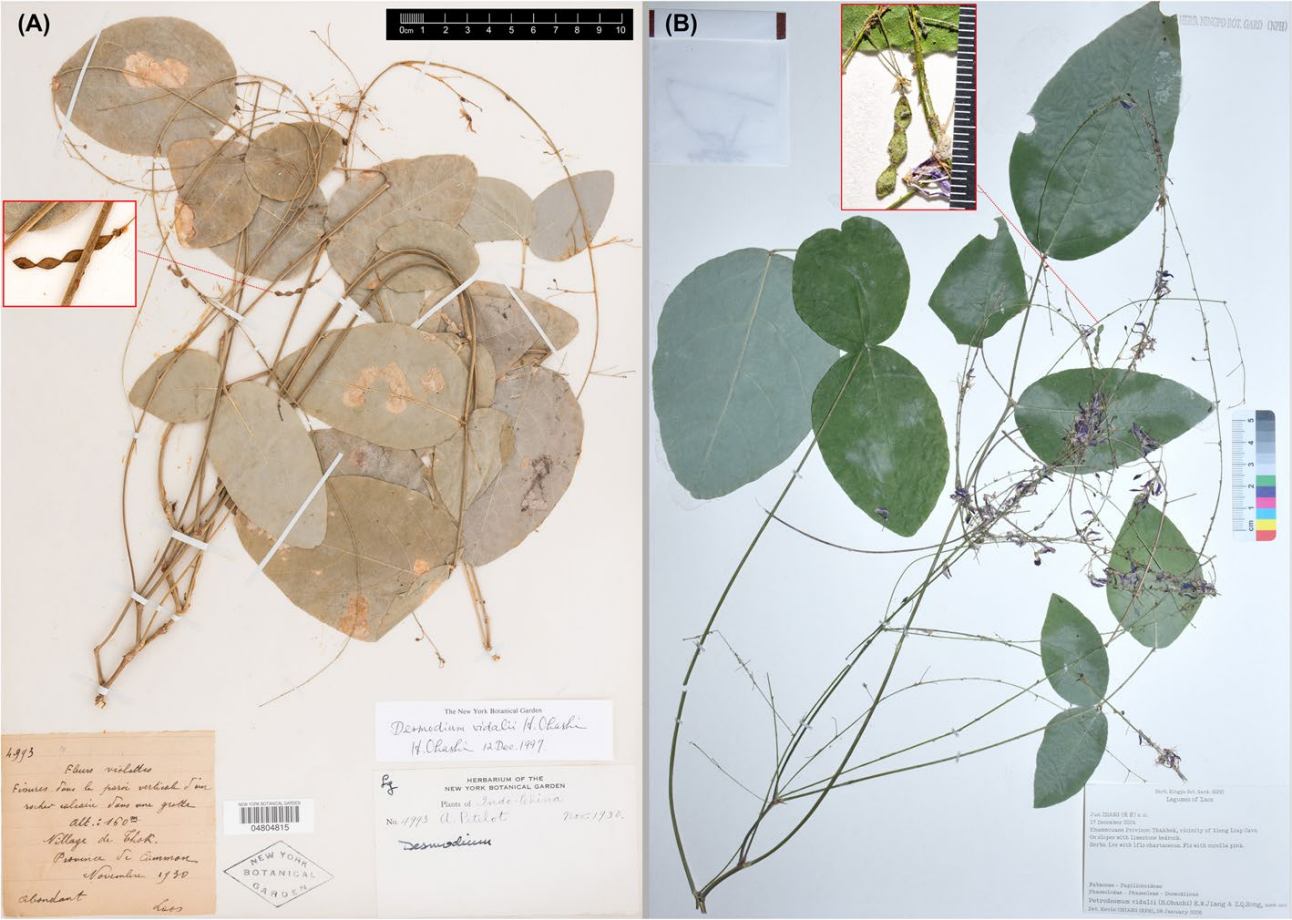
Specimens of *Grona vidalii* (basionym: *Desmodium vidalii*). **A** isotype (*H. Pételot 4993,* NY04804815, available at https://sweetgum.nybg.org/images3/4101/658/04804815.jpg, ©New York Botanical Garden); (**B**) DNA sampled specimen from the type locality (*J. Zhang s.n.,* NPH)

We examined all available herbarium specimens or high-resolution specimen images of the Asian species within the tribe Desmodieae deposited in the following herbaria: A, BKF, BM, E, HITBC, IBK, IBSC, K, L, NPH, NY, MO, US, P, PE, and U. These included the holotype (P02142472) and one isotype (NY04804815, Fig. 1A) of *Desmodium vidalii*, along with a DNA sampled collection [*J. Zhang s. n.* (NPH), Fig. 1B] from the type locality (Thok Village, Thakhek District, Khammouane Province, Laos) of this species, as well as specimens of related genera such as *Hylodesmum*, *Monarthrocarpus*, and *Grona*. Herbarium codes follow Index Herbariorum [38].

### Taxon sampling, sequencing, and assembly

To clarify the phylogenetic position of *Desmodium vidalii* within the tribe Desmodieae, we obtained the DNA data for a total of 254 samples (see Table S1), representing all 47 genera and 174 of 550 species (ca. 32%) in the tribe. Most samples were used in previous studies [5, 25–27, 29, 31, 32, 33]. Twenty-four samples were newly analyzed in this study, including one representative of *Desmodium vidalii*, 15 samples of *Hylodesmum*, two of *Monarthrocarpus*, three of *Desmodium*, and three of other genera. An additional five samples of *Mucuna* Adans. and one sample of *Haymondia* were selected as the outgroup based on previous studies [5, 11, 43].

Total DNA for the new samples was extracted from silica-gel-dried leaves or herbarium specimens using a modified CTAB method [3]. Nuclear ribosomal DNA and plastid sequences were obtained via a genome skimming approach [41]. Libraries were constructed and sequenced on an Illumina HiSeq X Ten platform (BGI, Wuhan, China). Subsequently, the sequences were assembled using GetOrganelle [6], annotated with PGA [35], and manually verified in Geneious v11.0.4 [9]. *Hylodesmum podocarpum* (DC.) H.Ohashi & R.R.Mill (MG867568) and *Trifolium repens* L. (MT735335) were used as reference genomes for the plastid and nrDNA annotation, respectively.

### Sequence alignment and phylogenetic analyses

We constructed two concatenated datasets: one from plastid DNA and one from nuclear ribosomal DNA (nrDNA). The plastid dataset comprised: (1) complete plastomes (including 81 protein-coding genes and 128 intergenic regions) from 43 samples, and (2) nine intergenic regions (including *trnK* intron, *ndhJ-trnL-trnF*, *trnT-trnL*, *trnG-trnS*, *trnQ-rps16*, *trnL-rpl32*, *rpl16* intron, *trnC-rpoB* and *ndhA* intron) from 150 samples. The nuclear dataset included: (1) nrDNA (including ETS, 18S, ITS, and 26S) from 62 samples, (2) ITS sequences from 150 samples, and (3) ETS sequences from the same 119 samples. Individual genomic regions were aligned separately using MAFFT v7.490 [8] under the L-INS-i algorithm. The plastid and nrDNA alignments were then processed with trimAl v1.5 [1] with the “automated1” setting to remove unreliable regions. All leading and trailing gaps were treated as missing data.

Phylogenetic relationships were inferred using Maximum likelihood (ML) analyses in IQ-TREE v3.0.1 [13]. The best-fit substitution model for each DNA region was selected automatically via ModelFinder based on the Bayesian Information Criterion. Branch support was assessed using the Shimodaira–Hasegawa approximate likelihood ratio test (SH-aLRT) with 1,000 replicates and the ultrafast bootstrap (UFboot) approximation with 1,000 replicates, with parameters set at “-alrt 1000 -bb 1000.” Clades with SH-aLRT ≥ 80% and UFboot ≥ 95% were considered well-supported.

To gain an independent perspective on phylogenetic relationships within Desmodieae, we reconstructed a nuclear phylogeny using a target-capture dataset targeting the Angiosperms353 low-copy nuclear gene set, following the framework established by Zuntini et al. [43]. The sampling included 21 genera from the tribe and two outgroup taxa. Target capture was performed using HybPiper v2.3.2 [7] with the mega353 target file (available at https://github.com/chrisjackson-pellicle/NewTargets/blob/master/mega353.fasta.zip, [12]). To ensure data quality, we removed genes shorter than 45% of the mean length or below 100 bp, as well as samples with fewer than 20% of genes recovered. Genes present in less than 20% of the samples were also excluded. Additionally, 32 genes identified as putative paralogs by HybPiper were discarded to mitigate phylogenetic noise from paralogy. The final dataset comprised 321 nuclear genes from 28 species, with leading and trailing gaps treated as missing data. Individual gene trees were inferred using the same maximum likelihood framework applied to the plastid dataset. These gene trees subsequently used as input for species tree inference using ASTRAL-III v5.7.8 [42], with nodal support assessed via local posterior probabilities (LPP). Following the criterion of Sayyari and Mirarab [36], clades with LPP > 0.9 were considered strongly supported.

## Results

### Morphological studies

Comparisons of *Grona vidalii* (previous *Desmodium vidalii*) with three morphologically similar genera (including *Grona*, *Hylodesmum*, and *Monarthrocarpus*) are summarized in Table 2. Among the examined characters, the short zigzag stems, leaves with an exceptionally long petiole and a long rachis of the terminal leaflet, and restriction to limestone habitats represent unique morphological and ecological features of *G. vidalii* within the tribe Desmodieae. These distinctive traits support its morphological distinction from all other genera in the tribe.

**Table 2:** Comparison among *Petrodesmum*, *Grona*, *Hylodesmum*, and *Monarthrocarpus*

Character	*Petrodesmum*	*Grona*	*Hylodesmum*	*Monarthrocarpus*
Habit	perennial herbs	subshrubs or herbs, sometimes shrubs	perennial herbs	herbaceous subshrubs
Stem	short, zigzag	erect or often ascending	erect or ascending	erect
Leaflet number	3	3 and/or 1	3, rarely 5–7	3 and/or 1
Petiole length	9–25 cm	0.5–5 cm	(1.5–)4–12 cm	2.5–5 cm
Principal lateral nerves	directly reaching margin	usually not reaching margin	directly reaching margin, or not	not reaching margin
Terminal leaflet rachis	3.5–7 cm long	0.3–1.2 cm long	(0.5–)1–2(–3) cm long	0.6–3 cm long
Inflorescence placement	terminal and axillary	terminal or terminal and axillary or very rarely leaf-opposed	terminal or terminal and axillary, sometimes arising from the base of main stem	terminal and axillary
Inflorescence type	lax-flowered, with 2–3 flowers per node	dense or lax-flowered, usually with 2–4 flowers per node	lax-flowered, with 2–5 flowers per node	lax-flowered, with 2 flowers per node
Inflorescence length	30–50 cm	1–20(–30) cm	15–80 cm	5–20 cm
Secondary bract	present	absent	present	absent
Androecium	diadelphous	diadelphous	monadelphous	diadelphous
Ovary	stipitate	mostly sessile	stipitate	stipitate
Pods	stipitate	sessile or stipitate	distinctly stipitate	stipitate
Pod article number	3–4	more than 3	2–5	1
Pod article shape	elliptic or obovate	semiorbicular to quadrangular, sometimes elliptic-oblong	semiorbicular or obliquely depressed, or very shallowly obtriangular	semilunar, narrowly rhombic, or dolabriform
Pod isthmus	isthmus less than 1/4 as broad as pod	isthmus 1/2–4/5 as broad as pod	isthmus less than 1/5 as broad as pod	absent
Pod sutures	lower and upper sutures deeply incised	upper suture straight, whereas lower suture shallowly or moderately incised	upper suture straight or shallowly undulate, whereas lower suture very deeply incised	upper suture straight or shallowly undulate, whereas lower suture very deeply incised
Seed	unknown	distinctly with rim-aril around hilum	without rim-aril around hilum	without rim-aril around hilum
Habitat	limestone habitat	thickets, grassy slopes, wastelands	forests, thickets, shaded places along streams	forests, thickets, shaded places along streams
Species included	*Grona vidalii*	49 species	13 species	2 species
Distribution	Central Laos	Asia, America, and Africa	Asia, America, and Africa	Asia and Madagascar
References	Ohashi [15], Ohashi and Ohashi [23]	Ohashi and Ohashi [19, 20, 21], Ohashi et al. [31, 32]	Ohashi [14], Ohashi and Mill [17], Song et al. [37]	Ohashi [14], Ohashi and Ohashi [18, 22]

### Phylogenetic relationships

**Fig. 2:**
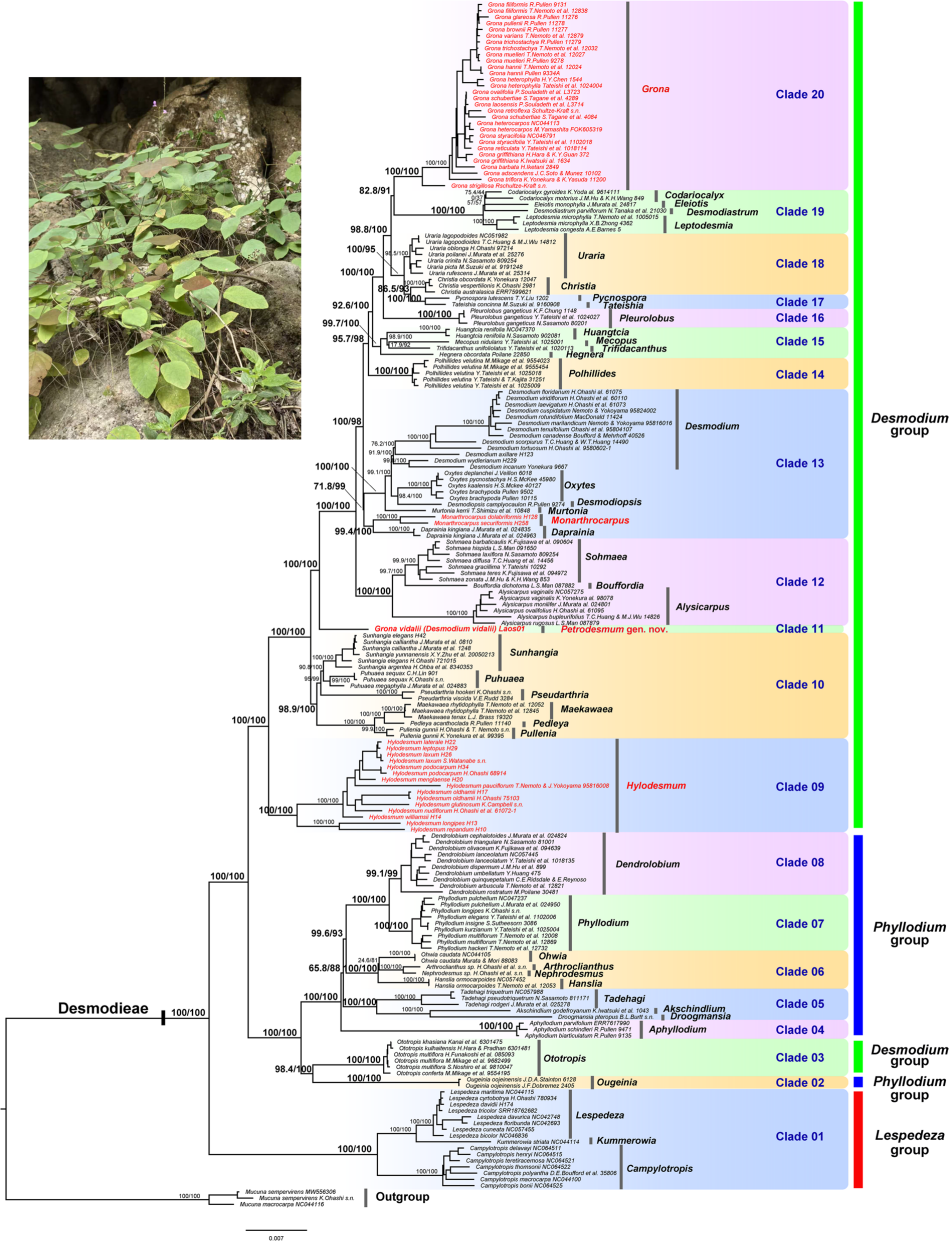
Maximum likelihood phylogeny of the tribe Desmodieae inferred from the concatenated plastid dataset (including 43 complete plastomes and nine plastid intergenic regions from 150 samples). Support values (SH-aLRT/UFboot) are shown on the branches. Taxa in red indicate *Grona vidalii* (with a field photograph in the upper left corner) and its three morphologically similar genera, *Hylodesmum, Monarthrocarpus* and *Grona*

**Fig. 3:**
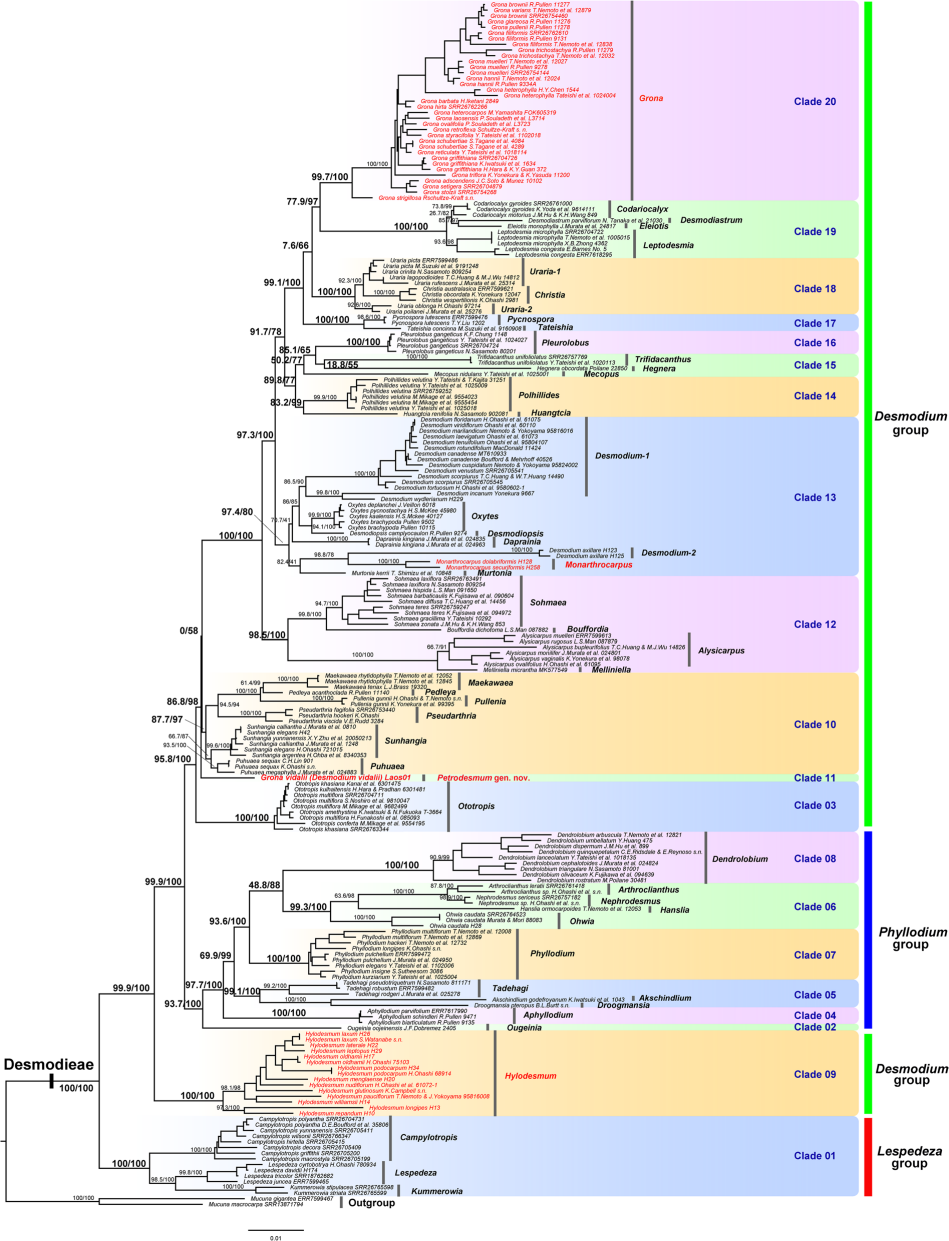
Maximum likelihood phylogeny of the tribe Desmodieae inferred from the concatenated nrDNA dataset (including nuclear ribosomal DNA for 62 samples, ITS sequences for 150 samples, and ETS sequences for 119 samples). Support values (SH-aLRT/UFboot) are shown on the branches. Taxa in red indicate *Grona vidalii* (*Desmodium vidalii*) and its three morphologically similar genera, *Hylodesmum, Monarthrocarpus* and *Grona*

**Fig. 4:**
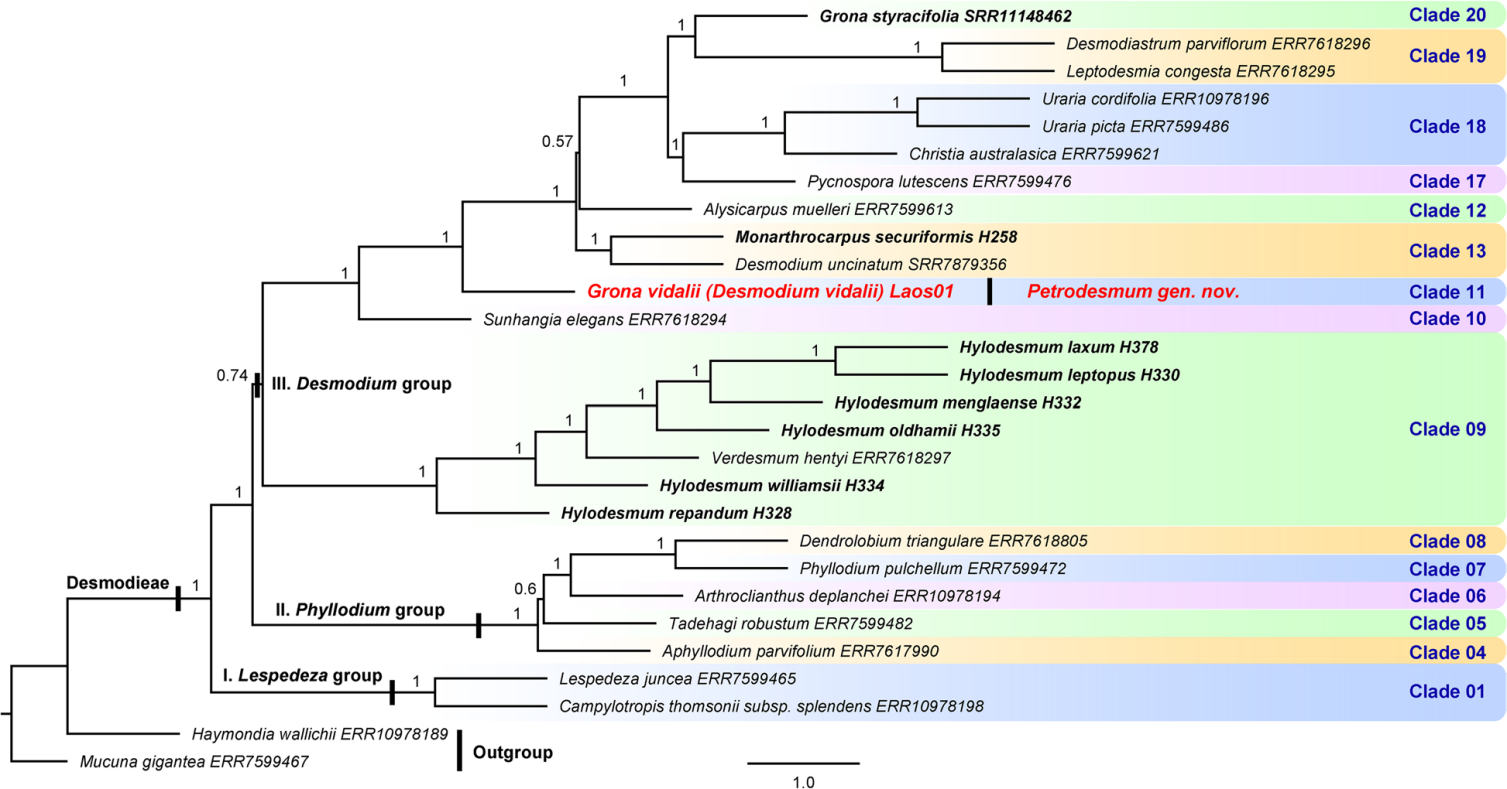
Species tree of Desmodieae based on 321 single-copy nuclear genes using ASTRAL-III v5.7.8. Numbers near branches are local posterior probabilities (LPP)

The phylogenetic relationships within Desmodieae, reconstructed using maximum likelihood (ML) analyses, are presented in Fig. 2 (plastid dataset: 43 complete plastomes and nine intergenic regions from 150 samples) and Fig. 3 (nrDNA dataset: 62 nuclear ribosomal DNA sequences, 150 ITS, and 119 ETS sequences). The species tree referred via the coalescent method is shown in Fig. 4 (nuclear dataset: 321 single-copy nuclear genes from 28 species). Overall, all three phylogenies recovered well-resolved intergeneric relationships, with the plastid tree featuring the most extensive taxon sampling and generally higher nodal support. The monophyly of the tribe Desmodieae (SH‑aLRT/UFBoot = 100/100 in Figs. 2 and 3; LPP = 1 in Fig. 4) and the *Lespedeza* group (100/100 in Figs. 2 and 3; LPP = 1 in Fig. 4) were consistently strongly supported across all three analyses. By contrast, the *Desmodium* group was resolved as non‑monophyletic in the plastid and nrDNA trees, whereas it appeared monophyletic in the nuclear tree, albeit with weak support (LPP = 0.74 in Fig. 4). The *Phyllodium* group received strong support for monophyly in the nrDNA (93.7/100 in Fig. 3) and nuclear trees (LPP = 1 in Fig. 4) but was recovered as paraphyletic in the plastid tree.

Comparative analysis of all three phylogenies identified 20 major clades (clades 01–20) within Desmodieae, although only 15 major clades were recovered from the nuclear tree owing to limited taxon sampling (Fig. 4). Nearly all clades exhibited consistency in generic composition between the plastid and nrDNA trees, with the exception of clades 14 and 15. *Huangtcia* H.Ohashi & K.Ohashi was resolved within clade 15 in the plastid tree (99.7/100 in Fig. 2) but was placed in clade 14 in the nrDNA tree (83.2/99 in Fig. 3), leading to compositional incongruence between these two clades. Additionally, most clades were strongly supported as monophyletic across all three phylogenies, except for clades 13, 15 and 18. Clade 15 was recovered as non-monophyletic in the nrDNA tree, yet strongly supported as monophyletic in the plastid tree (99.7/100 in Fig. 2). Clade 18 was recovered as non-monophyletic in the plastid tree but strongly supported as monophyletic in the nrDNA tree (100/100 in Fig. 3). Clade 13 was supported as monophyletic in both the plastid and nrDNA trees, albeit with relatively weak support (71.8/99 in Fig. 2; 97.4/80 in Fig. 3).

Most currently recognized genera were recovered as monophyletic, with the exception of *Uraria* Desv., *Desmodium*, and *Hylodesmum*. The former two genera were resolved as non-monophyletic in the nrDNA tree but received strong support for monophyly (98.5/100 for *Uraria* and 91.9/100 for *Desmodium* in Fig. 2) in the plastid tree. *Verdesmum* H.Ohashi & K.Ohashi was found to be nested within *Hylodesmum* (Fig. 4), rendering the latter non-monophyletic. Notably, *Grona vidalii* (previous *Desmodium vidalii*) forms an isolated lineage, Clade 11, which is distantly related to its three morphologically similar genera: *Grona* (Clade 20, with 24 of its 49 species sampled), *Hylodesmum* (Clade 09, with 12 of its 13 species sampled), and *Monarthrocarpus* (a member of Clade 13, with both species sampled). The phylogenetic position of this species within Desmodieae is consistent between the plastid and nuclear trees: in both phylogenies it is sister to the clade comprising Clades 12–20. This relationship is strongly supported in the nuclear species tree (LPP = 1 in Fig. 4) and in the plastid tree (100/100; Fig. 2), despite an unresolved relationship among Clade 11, Clade 10, and the clade comprising Clades 12–20 in the nrDNA tree (0/58 in Fig. 3).

## Discussion

Morphologically, *Desmodium vidalii* is considered intermediate among *Hylodesmum*, *Monarthrocarpus*, and *Grona*, having historically been placed in or near these three genera [15, 23]. Initially, Ohashi [15] assigned this species to *Desmodium* subg. *Podocarpium* (Benth.) H.Ohashi based on shared characters including a herbaceous habit, evergreen trifoliolate leaves, long lax inflorescences, and deeply constricted, stipitate, hooked-hairy pods. This subgenus was later segregated into the distinct genera *Hylodesmum* and *Monarthrocarpus*. The loments of *Hylodesmum* and *Monarthrocarpus* consist of semiorbicular, obliquely depressed, or very shallowly obtriangular articles, with sutures deeply incised abaxially and straight adaxially. In contrast, *D. vidalii* produces loments with depressed‑elliptic to obovate articles that have incised sutures on both sides. Furthermore, *D. vidalii* differs from *Hylodesmum* in several other morphological features, including the stamen type, leaf petiole and rachis length, and stem morphology (see Table 1 and the Diagnosis section below). *Monarthrocarpus* is characterized by inflorescences lacking secondary bracts, diadelphous stamens, 1-seeded pods, and semilunar, narrowly rhombic or dolabriform articles [18, 22]. Although *D. vidalii* also possesses diadelphous stamens, it diverges markedly from *Monarthrocarpus* in the three other reproductive characters mentioned above, as well as in several vegetative traits such as the principal lateral nerves, leaf petiole and rachis length, and stem morphology (Table 1). Ecologically, *Hylodesmum* and *Monarthrocarpus* typically occur in forest habitats [17], whereas *D. vidalii* grows in limestone rock‑wall fissures [23], see also Fig. 2).

*Desmodium vidalii* also shares morphological affinities with *Grona*, a genus recently resurrected from *Desmodium *sensu lato. *Grona* was originally established by Loureiro [10] based on *G. repens* Lour. from Vietnam, but this name was later rejected in favor of the conserved name *Desmodium* published in 1813 (see [39]). Molecular evidence later led Ohashi and Ohashi [19, 20, 21] to reinstate *Grona* as a distinct genus, separate from *Desmodium *sensu stricto (the latter now restricted to the Americas with a single African species [22]). Accordingly, Ohashi and Ohashi [23] transferred *D. vidalii* to *Grona* as *G. vidalii*, noting shared characters such as ebracteolate pseudoracemes, a 4‑lobed calyx (with the adaxial lobe bifid and the abaxial lobe longest), diadelphous stamens, absence of a disk, and 3–4‑jointed linear loments with depressed‑obovate articles and hooked hairs. Nevertheless, *G. vidalii* differs markedly from other *Grona* species in its zigzag stems, leaves with an exceptionally long petiole and prolonged terminal‑leaflet rachis, principal lateral nerves reaching the margin directly, long and laxly flowered inflorescences bearing secondary bracts, deeply constricted and distinctly stipitate pods, and strict restriction to limestone habitats (see Table 1, Figs. 1–2).

This morphologically intermediate position of *Grona vidalii* is strongly supported by our molecular phylogenetic analyses. Analyses of the plastid, nrDNA, and single-copy nuclear datasets consistently resolve *G. vidalii* as an isolated lineage (Clade 11, Figs. 2, 3, 4), phylogenetically positioned between *Hylodesmum* (Clade 09), *Monarthrocarpus* (a member of Clade 13), and *Grona* (Clade 20). Its phylogenetic placement within Desmodieae is congruent between the plastid and nuclear trees: in both phylogenies, it is sister to the clade comprising Clades 12–20 (Figs. 2, 4), despite an unresolved relationship among Clade 11 (*G. vidalii*), Clade 10, and the clade comprising Clades 12–20 in the nrDNA phylogeny (Fig. 3). We suspect that this uncertainty stems from the comparatively low number of informative characters in the nrDNA alignment, which contrasts with the robust signal recovered from the plastid and single-copy nuclear datasets. Taken together, the morphological and molecular evidence robustly supports that *G. vidalii* constitutes a distinct evolutionary lineage within Desmodieae. Neither retaining this species in *Grona*, transferring it to another genus (e.g., *Hylodesmum* or *Monarthrocarpus*), nor recognizing it as an informal lineage without rank, would adequately reflect its unique evolutionary history and morphological-ecological distinctiveness. We therefore propose to recognize this Laotian species, *Grona vidalii* (basionym: *Desmodium vidalii*), as a new genus, *Petrodesmum*. In Laos, the tribe Desmodieae comprises approximately 78 species and three subspecies, representing 29 distinct genera (including the new genus described in this study; see Table S2). To facilitate identification and to highlight the morphological distinctions between this new genus and all other genera occurring in the region, we have compiled a generic key to the Desmodieae of Laos (see below).

### Taxonomic treatment

#### Key to the genera of Desmodieae in Laos


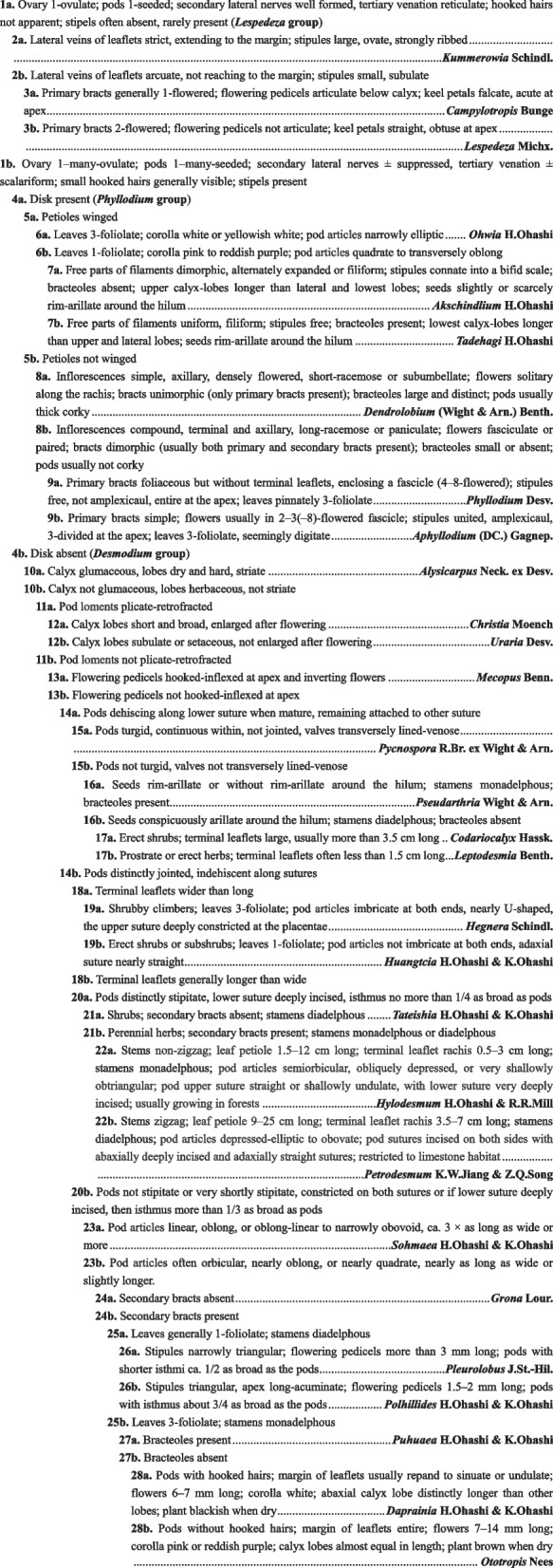


#### *Petrodesmum* K.W.Jiang & Z.Q.Song, gen. nov.

Type: *Petrodesmum vidalii* (H.Ohashi) K.W.Jiang & Z.Q.Song (basionym: *Desmodium vidalii* H.Ohashi).

Diagnosis: In morphology, the new genus *Petrodesmum* is most similar to *Hylodesmum* and *Grona* within the tribe Desmodieae. *Petrodesmum* is distinguished from *Hylodesmum* by having short zigzag stems (vs. erect or ascending, non-zigzag), an exceptionally long leaf petiole (9–25 cm vs. 1.5–12 cm), a long terminal leaflet rachis (3.5–7 cm vs. 0.5–3 cm), diadelphous stamens (vs. monadelphous), depressed‑elliptic to obovate pod articles (vs. semiorbicular, obliquely depressed, or very shallowly obtriangular), incised pod sutures on both sides with abaxially deeply incised and adaxially straight sutures (vs. upper suture straight or shallowly undulate, where lower suture very deeply incised), and limestone habitat (vs. forest habitat). *Petrodesmum* is distinguished from *Grona* by having short zigzag stems (vs. erect or often ascending, terete), an exceptionally long leaf petiole (9–25 cm vs. 0.5–5 cm), a long terminal leaflet rachis (3.5–7 cm vs. 0.3–1.2 cm), rather lax-flowered inflorescences (vs. dense or lax-flowered inflorescences), presence of secondary bract (vs. absence of secondary bract), depressed‑elliptic to obovate pod articles (vs. semiorbicular to quadrangular, sometimes elliptic-oblong), incised pod sutures on both sides with abaxially deeply incised and adaxially straight sutures (vs. upper suture straight, where lower suture shallowly or moderately incised), and limestone habitat (vs. thickets, grassy slopes, or wastelands).

Etymology: The generic name derives from *petros-* (Gk: rock) and *desmos* (Gk: chain, referring to an abbreviated form of *Desmodium*). The Chinese name is given as “石蚂蟥属” (shí mǎ huáng shǔ).

Distribution and habitat: Currently, this new genus is endemic to central Laos, and grows in the fissures of limestone rock walls.

#### *Petrodesmum vidalii* (H.Ohashi) K.W.Jiang & Z.Q.Song, comb. nov.

≡ *Desmodium vidalii* H.Ohashi, J. Jap. Bot. 67(6): 320. 1992 ≡ *Grona vidalii* (H.Ohashi) H.Ohashi & K.Ohashi, J. Jap. Bot. 95(1): 3. 2020. Type: Laos, Province de Camman [Khammouane Province], Thakhek District, village de Thok [Thok Village], “Fissures dans les parois verticales d'un rocher dans une grotte”, elev. 160 m, Nov. 1931, *H. Pételot 4993* (holotype: P02142472, photo!; isotype: NY04804815, photo!).

Description: Perennial herbs, 50–60 cm tall; stems generally simple, sometimes branched, ca. 10 cm long, zigzag, bent at each node, striate when dry, sparsely covered with spreading, minute, ± hooded hairs. Rootstocks ± woody, thickened. Stipules persistent, triangular-ovate, long-acuminate at apex, 5–8 × 2–4 mm, scarious, striate, glabrous adaxially, sparsely covered with spreading hairs abaxially, sparsely ciliate. Leaves evergreen, pinnately 3-foliolate, 17–32 cm long; petioles 9–25 cm long, indumentum as on stems but generally denser; rachis 3.5–7 cm, pubescent as on petioles; leaflets chartaceous, terminal one ovate to lanceolate-ovate, 7–13 × 4.5–12.5 cm, lateral ones slightly asymmetrical, 3.4–8.8 × 1.8–5.8 cm, adaxial surface green, sparsely to densely puberulent with ± appressed, minute, hooked hairs, abaxial surface greyish-green, densely puberulent with short, straight, erect, soft hairs, and rather sparsely mixed with longer, ± spreading hairs that are denser along veins and margins; midvein prominent abaxially, principal lateral veins 6–7 pairs, prominent abaxially, reaching the margin directly; reticulation prominent abaxially; petiolules 1.5–3 mm, moderately pubescent as on petioles; stipels linear-triangular, scarious, 1.5–2.5 mm, moderately ciliate. Inflorescences terminal and axillary, pseudoracemose, solitary to ± branched, long-pedunculate, lax-flowered, with 2–3 flowers at each node, 30–50 cm long; peduncles 8–30 cm long; axes sparsely to densely pubescent as on petioles. Pedicels 8–15 mm, pubescent as on axes. Primary and secondary bracts triangular, acute to acuminate at apex, 3.5–4 × ca. 1 mm, scarious, striate, sparsely pubescent on both surfaces, moderately ciliate, deciduous; bracteoles absent. Calyx funnel-form, 4-lobed above middle, pubescent abaxially with straight hairs and minute hooked hairs, glabrous adaxially; tube ca. 1.5 mm; upper lobe bifid at apex, ca. 1.5 mm; lateral lobes triangular, acuminate, 1.5–2 mm; lowest lobe longer than the laterals, 1.7–2.2 mm. Corolla pink or pale purplish-red; standard obovate, ca. 8 × 3 mm, rounded at apex, attenuate toward base, subsessile; wings narrowly falcate, ca. 8 × 1 mm, acute to acuminate at apex, claw ca. 1.5 mm; keel falcate, strongly incurved to ca. 90°, ca. 7 × 1.5 mm, acuminate at apex, claw ca. 1 mm long. Stamens 10, diadelphous, 9 + 1, ca. 7 mm, glabrous. Pistil shortly stipitate, 3–4-ovulate; style incurved. Pod a loment, shortly stipitate, 3–4-jointed; articles elliptic, ca. 4.5 × 2 mm, moderately to densely covered with uncinate hairs, reticulate, deeply constricted between the seeds with central isthmi; each isthmus less than 1/4 as broad as the pod. Seeds unknown.

Additional specimens examined: Laos, Khammouane Province, Thakhek, vicinity of Xieng Liap Cave, on slopes with limestone bedrock, 17 Dec. 2024, *J. Zhang s. n.* (NPH).

## Supplementary Information

Supplementary Material 1.

Supplementary Material 2.

## Data Availability

All of the data that support the findings of this study are available in the main text and Supporting information.
